# Release of Danger Signals during Ischemic Storage of the Liver: A Potential Marker of Organ Damage?

**DOI:** 10.1155/2010/436145

**Published:** 2010-12-21

**Authors:** Anding Liu, Hao Jin, Olaf Dirsch, Meihong Deng, Hai Huang, Martina Bröcker-Preuss, Uta Dahmen

**Affiliations:** ^1^Experimental Transplantation Surgery, Department of General, Visceral and Vascular Surgery, Friedrich Schiller University Jena, Drackendorfer Str.1, 07747 Jena, Germany; ^2^The Centre for Molecular Medicine, Shaoxing People's Hospital, 312000 Shaoxing, China; ^3^Department of General, Visceral and Transplantation Surgery, University Hospital Essen, University of Duisburg and Essen, 45122 Essen, Germany; ^4^Institute of Pathology, University Hospital Jena, 07747 Jena, Germany; ^5^Department of Clinical Chemistry, Clinic of Endocrinology, University Hospital Essen, University of Duisburg and Essen, 45122 Essen, Germany

## Abstract

Liver grafts suffer from unavoidable injury due to ischemia and manipulation before implantation. Danger signals such as high-mobility group box -1(HMGB1) and macrophage migration inhibitory factor (MIF) play a pivotal role in the immune response. We characterized the kinetics of their release into the effluent during cold/warm ischemia and additional manipulation-induced mechanical damage. Furthermore, we evaluated the relationship between HMGB1/MIF release and ischemic/mechanical damage. Liver enzymes and protein in the effluent increased with increasing ischemia time. HMGB1/MIF- release correlated with the extent of hepatocellular injury. With increasing ischemia time and damage, HMGB1 was translocated from the nucleus to the cytoplasma as indicated by weak nuclear and strong cytoplasmic staining. Enhancement of liver injury by mechanical damage was indicated by an earlier HMGB1 translocation into the cytoplasm and earlier release of danger signals into the effluent. Our results suggest that determination of HMGB1 and MIF reflects the extent of ischemic injury. Furthermore, HMGB1and MIF are more sensitive than liver enzymes to detect the additional mechanical damage inflicted on the organ graft during surgical manipulation.

## 1. Introduction

The term “transplantation injury” describes the combination of all damaging events inflicted on the graft during the transplantation procedure. The explantation injury is related to the extent of mechanical stress during organ manipulation and to some degree is unavoidable during the removal of the organ from the donor. Mechanical stress may vary depending on the individual surgeon as well as the individual patient. Ischemia injury is induced by the combination of ischemia and cold ischemia. Reperfusion injury and the mainly mechanical implantation injury occur during organ implantation. 

Damage inflicted on cells leads to release of danger signals from dying cells or activated immune cells [1]. Danger signals translate a damage into a molecular event, which triggers the innate and adaptive immune response [1, 2]. 

Whatever inflicts damage on the body is detrimental. This is the basic idea of the danger model of immunology which was introduced by Matzinger in 1994 [3]. According to the danger model in immunology, only antigen presenting cells activated by cellular alarm signals from distressed cells are able to initiate an immune response in an organism [4, 5]. Alarm signals, such as heat shock protein 70 [6] and high mobility group box protein 1 (HMGB1), are endogenous danger signals [7, 8]. Danger signals form Damage-Associated Molecular Patterns (DAMPs) [9]. DAMPs are detected by pattern recognition receptors, orchestrating the inflammatory and immunologic response [10, 11]. The role of danger signals for the evaluation of liver graft damage remains unclear. Quantification of danger signals, such as HMGB1 and macrophage migration inhibitory factor (MIF) prior to implantation of a graft, may be helpful in the quantification of preimplantation organ damage and could serve as an indicator of organ quality, especially in marginal grafts.

## 2. Materials and Methods

### 2.1. Experimental Design

The experiments were designed to investigate the effect of warm and cold ischemia as well as mechanical damage on the intracellular location and the release of damage markers—HMGB1 and MIF—into the saline solution. Warm ischemic injury was induced by storing the liver at 37°C. Cold ischemic injury was induced by storing the liver at 4°C. Additional mechanical damage was inflicted by placing a weight on the liver graft. Two rat strains were used to test whether the release is strain independent (Table 1).

### 2.2. Animals

Male inbred Lewis and BN rats (Central Animal Facility of the University Hospital Essen), with a weight at approximately 300–350 g, were used in this study. All animals were housed under standard animal care conditions and had free access to water and rat chow ad libitum. All procedures were carried out according to the German Animal Welfare Legislation. Animal experiments were approved by the Bezirksregierung Düsseldorf.

### 2.3. Surgical Procedures

Surgical procedures were performed under inhalation anesthesia with isoflurane (Sigma-Aldrich, St. Louis, USA), (isoflurane concentration 3%, oxygen flow 0,5 l/min). After opening the abdomen with a transversal incision, the liver was freed from its ligaments and flushed with cold saline solution. 

In the mechanical stress groups, livers were subjected to mechanical stress by placing a metal weight of 100 g repeatedly (10 times) for 1 min on the liver in situ prior to explantation.

Infrahepatic vena cava and portal vein were cannulated with 12 G and 14 G catheters, respectively. Cannulated livers were placed in the incubator (37°C) or refrigerator (4°C). At defined intervals time (30 min warm ischemia, 1 h cold ischemia), the livers were flushed with saline (4°C) at a constant pressure of 10 cm H_2_O through the portal vein, and 1.5 ml effluent was collected from the infrahepatic vena cava. Protease inhibitors (1 ug/ml aprotinin, 1 ug/ml leupeptin, 1 ug/ml pepstatin, 1 mM PMSF 1 mM NaF 1 mM Na3VO4) (Sigma-Aldrich, St. Louis, USA) were added to the effluent sample immediately after collection. Samples were centrifuged thereafter to remove red blood cells.

### 2.4. Liver Damage Assessment

To assess hepatocellular injury following cold or warm ischemia, aspartate aminotransferase (AST) and alanine transaminase (ALT) were measured in the effluent using an Automated Chemical Analyzer (Bayer; Leverkusen, Germany).

### 2.5. Histopathology

Liver tissue was fixed in 4.5% buffered formalin for at least 24 h. Paraffin embedding was performed using standard techniques. Sections (4 *μ*m) were cut and stained with Hematoxylin-Eosin. Histological evaluation focused on signs of cellular damage, such as vacuolization, cell dissociation, cell swelling, and necrosis.

### 2.6. Gel Electrophoresis and Western Blotting

Effluent samples were boiled for 10 min in 1x loading buffer (0,06 M Tris-HCl, 5% SDS, 0.3% bromophenol blue, 10% glycerol, and 0,1 M Dithiothreitol) and were separated on 1,5 mm 12% mini gels by SDS-PAGE. Proteins were transferred to polyvinylidene difluoride (PVDF) membranes (GE Healthcare, Buckinghamshire, UK) using a tank transfer unit (Hoefer, San Francisco, USA). Membranes were blocked using 5% milk solution (5% nonfat milk powder, 0.1%Tween 20 in PBS) for 1 h at room temperature. Primary antibodies (anti-HMGB1 polyclonal antibody, 1 : 1000, Abcam, Cambridge, UK; anti-MIF polyclonal antibody, 1 : 3000, Abcam, Cambridge, UK) were added to the membranes for 1 h at room temperature. Membranes were probed with secondary goat antirabbit or donkey antigoat antibodies conjugated to horseradish peroxidise (Abcam, Cambridge, UK). Detection was performed employing the Lumi-Light Western Blotting Substrate (Roche Applied Science, Mannheim, Germany) and high sensitivity films (GE Healthcare, Buckinghamshire, UK). Digitalization of films was performed using a scanner (Epson V750, Nagano, Japan). Quantification of band density was performed using Image J 1.40 G (NIH, Bethesda, USA). A standard curve covering a range from 0 to 2000 ng/ml was generated using recombinant HMGB1 (Sigma-Aldrich, St. Louis, USA) to calculate the concentration of HMGB1 in the effluent. In contrast, the result of the quantification for MIF was expressed in arbitrary units, since no purified recombinant MIF was available.

### 2.7. Silver Staining

Following electrophoresis, acrylamide gels were incubated in fixing buffer for 1 h (40% ethanol, 10% acetic acid). Subsequently, gels were submersed in a 5% ethanol-5% acetic acid solutions overnight. Gels were then rinsed in distilled water for 5 min and soaked in 10% glutaraldehyde solution (Sigma-Aldrich, St. Louis, USA) for 30 min at room temperature. Glutaraldehyde was removed by washing with deionized water. Gels were incubated in freshly prepared 0.1% ammoniacal silver nitrate solution (Merck, Darmstadt, Germany) for 30 min. After incubation, gels were again briefly washed in distilled water and then incubated in developing solution buffer (0.01% formaldehyde and 0.01% citric acid) for 5 to 10 min. The reaction was stopped by the addition of 5% acetic acid solution to the gels.

### 2.8. Immunohistochemical Staining

For the immunohistochemical detection of HMGB1, antigen retrieval was performed in a water bath using citrate-EDTA buffer (10 mM Citric Acid, 2 mM EDTA, 0.05% Tween 20, pH 6.2) for 20 mins at 100°C. Nonspecific protein binding was blocked using 100 ul serum-free blocking buffer (Dako, Glostrup, Denmark). Slides were washed 3 times with PBS. Sections were incubated with diluted (1/500) polyclonal rabbit anti-HMGB1 antibody (Abcam, Cambridge, UK) for 1 hour at room temperature. The slides were rinsed with PBS, and detection was performed using PowerVision goat-anti-Rabbit-AP (ImmunoLogic, Duiven, Netherlands) employing Fast-Red (Dako, Glostrup, Denmark) as substrate. Sections were counterstained with Hematoxylin for 5 mins. The staining was documented using a digital camera (Olympus, Tokyo, Japan) mounted on a microscope (Leica,Wetzlar, Germany) at a magnification of 200x. Three pictures—one from each lobular zone [12]—were selected randomly to analyze HMGB1 staining. The percentage of hepatocytes with only nuclear HMGB1 staining and only cytoplasmic HMGB1 staining out of the total number of hepatocytes in the three pictures taken (800–1000 cells) was calculated.

### 2.9. Statistical Analysis

Data are expressed as mean ± SD. Differences between groups were evaluated for significance by one-way ANOVA analysis. Bivariate correlations were tested with Spearman's rank correlation. All tests were performed using SigmaStat v3.5 (Systat-Software, Erkrath, Germany). A *P*-value below.05 was considered statistically significant.

## 3. Results

### 3.1. Proteins in the Effluent Increased with Ischemia Time and Subsequent to Mechanical Stress

**Figure 1:**
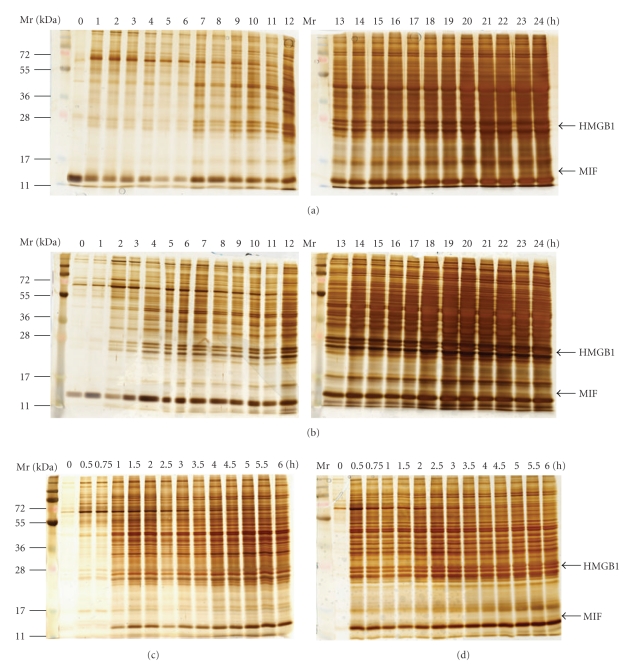
Release of proteins during ischemic storage of liver. Release of proteins into the effluent collected at defined intervals within after 24 h cold ischemia or 6 h warm ischemia was visualized by silver staining after electrophoresis. The concentration of total protein increased substantially within the first 30/60 min of warm/cold ischemia. Arrows indicated the positions of HMGB1 and MIF. (a) Cold ischemia; (b) mechanical stress plus cold ischemia; (c) warm ischemia, (d) mechanical stress plus warm ischemia.

Damage to the graft leads to the release of an array of proteins, such as liver enzymes, danger signals, and others. Total protein in the effluent was taken as a parameter reflecting the release of proteins into the ischemia solution and thereby indicating damage to the graft. For a qualitative assessment of the change in protein composition in the effluent caused by explantation and ischemia injury, silver staining was performed after gel electrophoresis of the effluent samples. Protein release into the effluent started after 1/0.5 h of cold/warm ischemia and increased with increasing ischemia time (Figures 1(a) and 1(c)). Within the array of proteins released into the effluent, we identified one protein with a molecular weight of about 28-kDa corresponding to the molecular weight of HMGB1 and another protein with a molecular weight of about 13-kDa corresponding to the molecular weight of MIF. We confirmed by immunoblotting that the bands with the molecular weight corresponding to HMGB1 and MIF showed a positive signal for the respective antibody. Additional mechanical damage enhanced proteins release (Figures 1(b) and 1(d)). Effluent acquired from BN livers showed a similar kinetic and staining pattern as effluent taken from Lewis livers (data not shown).

### 3.2. Liver Injury Increased with Ischemia Time and Mechanical Stress

**Figure 2:**
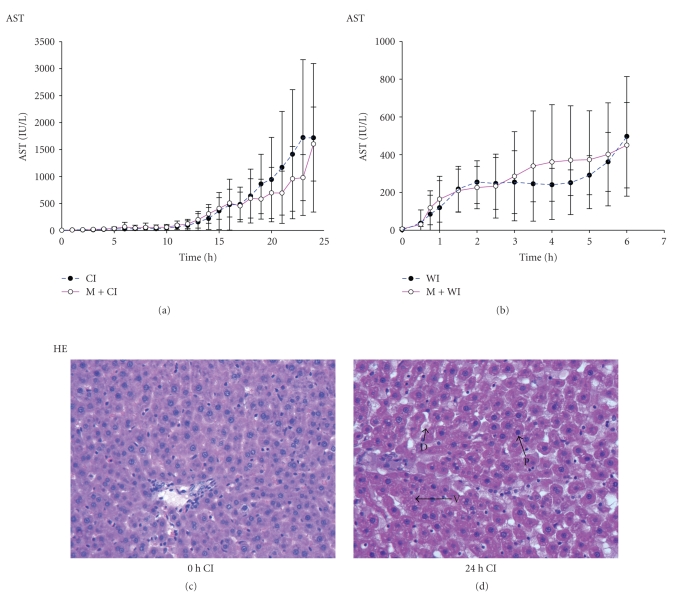
Liver damage during ischemic storage. Liver enzymes in the effluent increased gradually in parallel to the length of warm/cold ischemia time. Mean total liver enzymes values were higher, but not significantly (*P* > .05) ((a) and (b)). Liver injury was confirmed by liver histology (original magnification, 200x). Progressive changes in hepatocyte morphology such as cell dissociation (D), cytoplasmic vacuolization (V), and nuclear pycnosis (P) did increase over ischemia time. (c) 0 h cold ischemia (CI) liver tissue; (d) 24 h CI liver tissue.

To determine the extent of hepatocellular injury in relation to the explantation and ischemia period, AST and ALT were measured in the effluent. AST was undetectable immediately after explantation and gradually increased with time during warm or cold ischemia (Figures 2(a) and 2(b)). Moderate release of liver enzymes (mean >10 U/L) was observed after 0.5 h/2 h of warm/cold ischemia. Substantial release (mean >100 U/L) occurred after 1 h/13 h hours of warm/cold ischemia. AST release occurred earlier (0.5–1.5 hr/1–12 hr warm/cold ischemia) and reached higher, albeit not significantly higher (*P* > .05) levels when grafts were subjected to additional mechanical stress during explantation. Similar results were obtained for ALT and were not affected by the rat strain (data not shown).

Liver histology confirmed that hepatic damage increased with ischemia time. H&E staining showed normal hepatic morphology at early time points (0 h, 4 h cold ischemia, 0 h, 0,5 h warm ischemia, resp.) (Figure 2(c)). Vacuolization of cytoplasm and fragmentation of hepatocytcellular nuclei as well as hepatocyte dissociation occurred after 1, 8 h of warm/cold ischemia, respectively, and became prominent with increasing ischemia time. Progressive changes in cell morphology, such as cell swelling, cytoplasmic vacuolization, and nuclear fragmentation, were observed between 1 hr and 6 hr of warm ischemia, and similar results were obtained when extending cold ischemia time from 12 hr to 24 hr (Figure 2(d)).

### 3.3. Shift of HMGB1 Staining Pattern in Hepatocytes upon Extended Cold/Warm Ischemia

**Figure 3:**
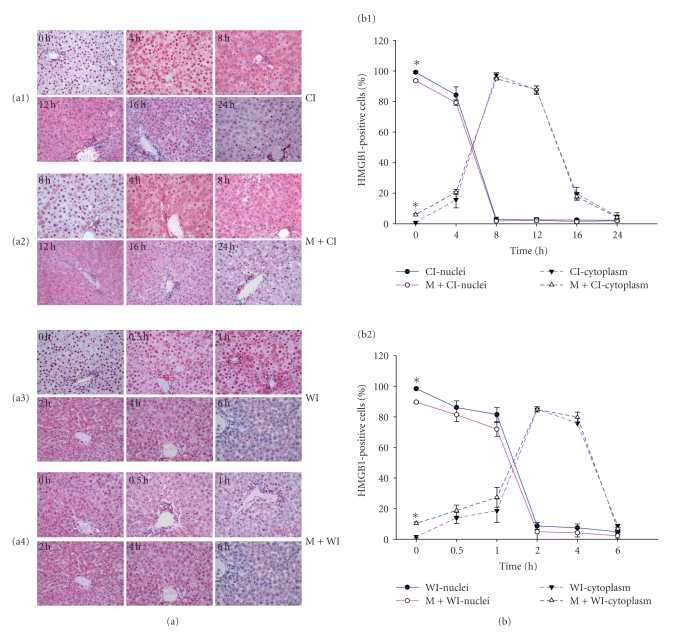
Immunohistochemical analysis of HMGB1 expression in rat livers following cold/warm ischemia. Staining pattern changed upon ischemia and mechanical stress (a). (a1) cold ischemia (CI), (a2) mechanical stress plus cold ischemia (M+CI), (a3) warm ischemia (WI), (a4) mechanical stress plus warm ischemia (M+WI). Relative frequency of hepatocytes with nuclear and cytoplasmic staining (b). In normal liver, only single hepatocytes showed cytoplasmic staining (<1%). The relative frequency increased with ischemia time and reached a peak after 2 h/8 h (85%/95%) of warm/cold ischemia and decreased continuously thereafter. Mechanical stress inflicted on the liver during explantation increased HMGB1 translocation (*P* < .05 versus without mechanical stress groups). Data shown are representative of all tissue samples analyzed at a magnification of 200x. *P < .05.

Immunohistochemical staining was performed to observe the intra-cellular distribution of HMGB1 in hepatocytes undergoing cold and warm ischemia only (Figures 3(a1) and 3(a3)) as well as additional mechanical injury (Figures 3(a2) and 3(a4)). The percentage of hepatocytes with nuclear HMGB1 staining, cytoplasmic HMGB1 staining, respectively, was calculated (Figure 3(b)). Immunoreactivity to HMGB1 was found in the nuclei of hepatocytes in normal livers (0 hr). Single hepatocytes (about 1%) showed cytoplasmic staining. When extending warm/cold ischemia to 2/8 hours, the proportion of cells with strong cytoplasmic (>85% in warm/cold ischemia) and weak or no nuclear staining increased substantially. When extending the ischemic time further to 6/24 hours of warm/cold ischemia, nearly no nuclear staining (<5% in warm/cold ischemia) and only weak or no cytoplasmic staining were detected. 

### 3.4. Mechanical Stress Associated with HMGB1 Translocation

**Figure 4:**
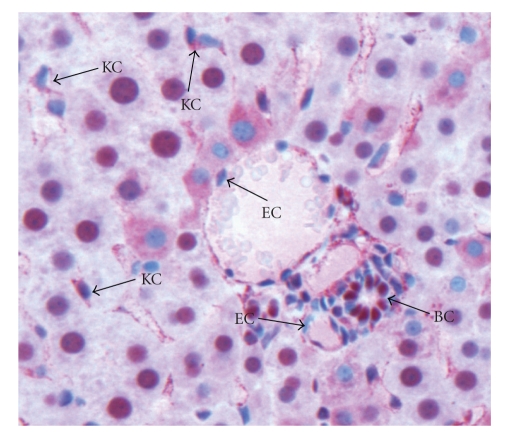
HMGB1-staining patterns in non-parenchymal cells. Kupffer cells (KCs) presented with weak nuclear staining but strong cytoplasmic staining. Vascular endothelial cells (ECs) were negative for HMGB1, and biliary epithelial cells (BC) were strongly HMGB1 positive during ischemia process (200x).

The percentage of hepatocytes with HMGB1 translocation to the cytoplasma indicated by the weak nuclear and strong cytoplasmic staining significantly increased immediately after mechanical stress to approximately 10%. In comparison to livers without mechanical stress this difference was statistically significant (*P* < .001). Nonparenchymal liver cells play an important role in the pathophysiology of warm/cold ischemia. Activated Kupffer cells have been identified as a critical source of HMGB1 and MIF. Kupffer cells and sinusoidal endothelial cells presented with weak nuclear staining and strong cytoplasmic staining immediately after explantation (Figure 4). In contrast to the staining pattern in hepatocytes, the staining pattern in vascular endothelial cells and biliary epithelial cells remained constant. Vascular endothelial cells were negative for HMGB1 at all time points. Biliary epithelial cells were strongly HMGB1 positive during the ischemia process throughout the observation period (Figure 4).

### 3.5. HMGB1 and MIF Release into the Effluent Is Dependent on the Extent of Damage

**Figure 5:**
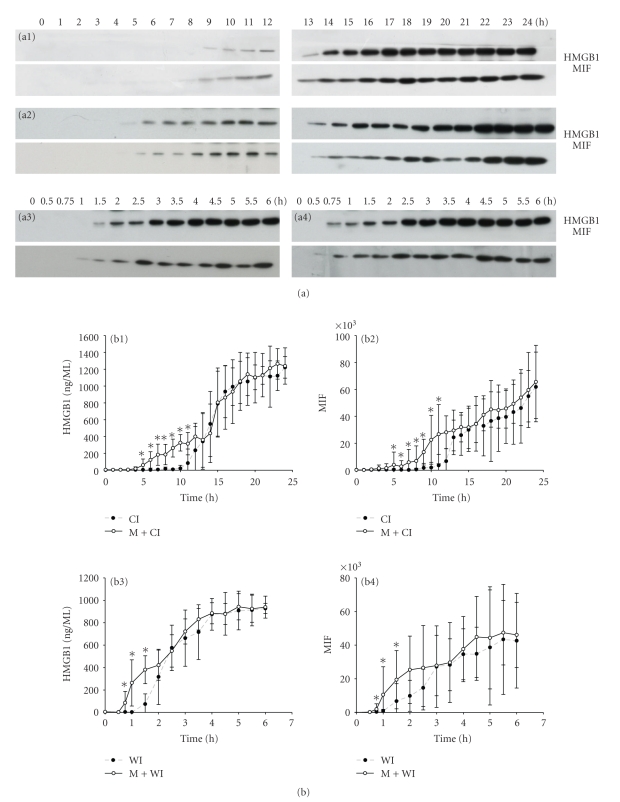
Release of danger signals during ischemic storage of liver. Effluent obtained after flushing the liver in half-hourly or hourly intervals for either 24 or 6 h was assessed for HMGB1 and MIF using Western blot (a). Release of both danger signals, HMGB1 and MIF, was detected by Western blot. (a1) Cold ischemia; (a2) mechanical stress plus cold ischemia; (a3) warm ischemia; (a4) mechanical stress plus warm ischemia. Band density was assessed using Image J program and expressed as ng/ml (HMBG1) or arbitrary units (MIF) (b). Danger signals were released significantly earlier in both groups subjected to mechanical stress compared with ischemia only group (*P* = .0027; *P* = .002, resp.). At earlier time points (0.75–1.5 h/5–11 h in warm/cold ischemic ischemia), the relative concentration of HMGB1 and MIF in the effluent was also significantly higher compared with groups subjected to ischemia only (*P < .05).

To determine whether HMGB1 and MIF were released and associated with hepatocellular injury, Western blot analysis was performed on effluent samples obtained at defined time points during the ischemia period. HMGB1 as well as MIF release was detected as early as 10.8 hr (8 hr–12 hr) cold ischemia or 1.9 hr (1.5 hr–2.5 hr) warm ischemia and increased gradually (Figure 5(a)). Mechanical damage induced by 10 minutes of weight stress during explantation followed by either warm or cold ischemia drastically increased the release of HMGB1 and MIF into the effluent. Band density was assessed using Image J and expressed as ng/ml (HMGB1) or arbitrary units (MIF) (Figure 5(b)). In mechanical stress groups, HMGB1 was already detected after 0.96 hr (0.75–1.5 hr) warm or 6.50 hr (5–8 hr) cold ischemia, respectively, which was significantly earlier than in groups subjected to ischemic injury only (*P* = .0027, *P* = .0020, resp.). 

At these time points (0.75–1.5 h/5–11 h in warm/cold ischemic ischemia), the relative concentration of HMGB1 in the effluent was also significantly higher compared with groups subjected to ischemia only (*P* < .05). MIF release, also detected by Western blot, followed a similar kinetic as HMGB1 release and was also released significantly higher upon mechanical stress compared to ischemia only. In contrast, additional mechanical stress did not lead to a significantly higher release of liver enzymes into the effluent compared with groups subjected to ischemia only (*P* > .05).

 A similar release pattern of HMGB1 was also observed in explanted BN livers, which were subjected to 24 h cold ischemia. These data indicated that HMGB1 was released in a strain-independent manner during ischemia of liver (data not shown).

### 3.6. AST and ALT as well as HMGB1 and MIF Show a Similar Release Pattern

**Figure 6:**
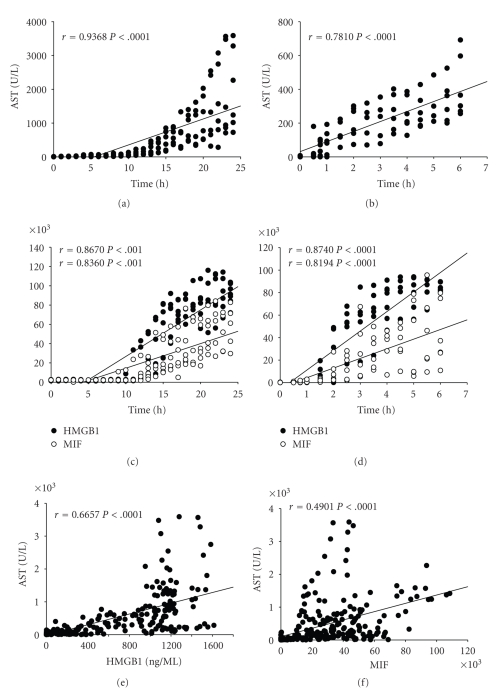
Correlation of Effluent HMGB1 and MIF with liver enzymes (AST) and ischemic time. Effluent AST positively correlated with cold or warm ischemic time ((a) and (b)), respectively, (*P* < .0001). For HMGB1, the values represent the density of band using Image J program ((c) and (d)), or the values represent the concentration quantified by recombinant standard (e). For MIF, the values represent the density of band using Image J program. Effluent HMGB1 and MIF levels correlated moderately with ischemic time ((c) and (d); *P* < .0001) and effluent AST ((e) and (f); *P* < .0001).

We determined the correlation between the release of hepatic enzymes and the length of the ischemic time. To determine this correlation, data from cold and warm ischemia without additional mechanical damage were used. AST positively correlated with cold or warm ischemic time (*r* = .9368, *P* < .0001 and *r* = .7810, *P* < .0001, resp., (Figures 6(a) and 6(b)). Evaluation of the correlation between the release of danger signals and ischemic time revealed a moderate correlation of HMGB1 and MIF release into the effluent with the length of cold (*r* = .8670, *P* < .0001 and *r* = .8630, *P* < .0001, resp.) and warm (*r* = .8740, *P* < .0001 and *r* = .8194, *P* < .0001, resp., Figures 6(c) and 6(d)) ischemic time. 

Calculating the correlation between HMGB1 release and liver enzymes, respectively, effluent HMGB1 moderately correlated with AST in the effluent (*r* = .6657, *P* < .0001) (Figure 6(e)). MIF, and AST release also moderately correlated (*r* = .4901, *P* < .0001) (Figure 6(f)). Similar results were obtained for ALT (data not shown). HMGB1 release from BN livers was moderately correlated to the release of liver enzymes (data not shown).

## 4. Discussion

In a previous rat liver transplantation study, we demonstrated that all rats died within 48 h after liver transplantation when the cold ischemia time in saline was prolonged to 10 h. Nearly 50% died once a cold ischemia time of 9 h was employed. Cold ischemia of the graft for a period under 9 h was associated with 100% survival (paper submitted for publication). A marked increase in AST, ALT as well as HMGB1, MIF was observed after 9–10 h of ischemia, the same length of graft ischemia time that led to decreased animal survival.

HMGB1 release following ischemic injury of the liver was observed in clinical as well as in experimental studies. HMGB1 has been implicated in ischemia/reperfusion injury of the liver [13], kidney [14], heart [15], and brain [16]. Ilmakunnas et al. were the first to evaluate the danger signal HMGB1 as a marker for hepatocellular injury. In their study, HMGB1 was undetectable in the systemic circulation before human liver transplantation, and the peak value appeared 10 minutes after portal vein declamping and showed moderate correlation with AST. They concluded that HMGB1 released from a human liver graft could be used as a marker of liver injury [17]. Tsung observed in a mouse ischemia/reperfusion model that HMGB1 protein expression in the liver increased with time up to 24 h following 60 min of warm ischemia of the liver [18], suggesting an active production of HMGB1 in addition to a cell damage-associated release. 

Extracellular HMGB1 is a mediator of inflammation [1, 19–21]. Before HMGB1 is released from cells, HMGB1 is translocated from the nucleus into the cytoplasm. Translocation is associated with acetylation [22], phosphorylation [23], and methylation [24] of HMGB1. Once released, HMGB1 exerts biologic effects through its receptors. Several receptors have been implicated in HMGB1 signalling, including the receptor for advanced glycation end product (RAGE) and Toll-like family receptors, such as Toll-like receptor 4(TLR4); Toll-like receptor 2(TLR2), and Toll-like receptor 9(TLR9) [25, 26]. Signalling of these receptors induces production and release of inflammatory cytokines by immune cells. In our study, we found that HMGB1 was released from liver cells after prolonged warm/cold ischemia. HMGB1 release to the extracellular space may be governed by two different mechanisms [27]. One mechanism is an active secretion process. This mechanism is employed by immune cells; HMGB1 can be actively secreted by activated macrophages, NK cells, and mature myeloid DCs [22, 28, 29]. A second mechanism is a passive release process. HMGB1 is released when cells undergo necrosis [30–35]. In this study, HMGB1 translocated from the hepatocellular nucleus to the cytoplasma and also to the extracellular space. The kinetic of the HMGB1 translocation was in parallel to the overall injury of the organ graft as demonstrated by a release of liver damage markers (AST and ALT) as well as an increase in proteins in the effluent as demonstrated by a silver stain in a polyacrylamide gel. Translocation to the cytoplasma was seen prior to the release into the effluent. Loss of cytoplasmic HMGB1 paralleled the detection in the effluent; supporting the hypothesis HMGB1 was indeed released by damaged hepatocytes. 

MIF release following ischemic injury of the liver was observed in clinical as well as in experimental studies. MIF is another well-known cytokine that is released from immune cells and plays an important role in inflammatory diseases [36–39]. It has been reported that MIF is constitutively expressed in the hepatocytes [40]. In contrast to most cytokines, MIF is stored in intracellular pools and secreted immediately by nonconventional protein-secretion pathway before de novo protein synthesis [41]. MIF was released by macrophages after stimulation with Lipopolysaccharide (LPS), Tumor necrosis factor-alpha (TNF-*α*), or Interferon-gamma (IFN-*γ*) [37, 41]. MIF level was elevated in tissue and serum in a mouse sepsis model [37] and also increased in the serum of patients with septic shock [42]. It has been reported that plasma MIF is significantly elevated in patients after liver resection, indicating that MIF has a role in mediation of systemic inflammatory response after surgery injury [43]. A rapidly growing body of evidence supports that MIF is involved in the inflammatory cascade and ischemia/reperfusion injury [44, 45]. It was reported that liver I/R injury causes the expression of MIF [46], and anti-MIF antibody attenuates hepatic injury in a mice endotoxin-induced fatal hepatic failure model. In this study, the amount of MIF was significantly increased after prolonged warm/cold ischemia. Liver non-parenchymal cells, including Kupffer cells and the sinusoidal endothelial cells, are sensitive to ischemia. MIF mRNA was increased immediately in livers during cold ischemia (data not included). It appears likely that MIF was mainly actively released from liver non-parenchymal cells.

HMGB1 release subsequent to mechanical trauma was described in several clinical and experimental papers. Peltz et al. reported that plasma HMGB1 increased significantly within 1hr in patients with blunt or penetrating trauma and a severity score greater than or equal to 15. Peak levels occurred from 2 to 6 hr after injury [47]. Using an experimental model, Levy et al. observed that HMGB1 was elevated in serum 1h after peripheral injury consisting of a bone fracture in mice [48]. 

MIF could be also an important cytokine in injury caused by trauma. Jeschke et al. reported that the amount of MIF in serum was significantly increased in rat thermal trauma models [49]. MIF gene expression and protein levels were significantly increased in murine and canine acute lung injury models. Serum level of MIF was also significantly elevated in patients with sepsis induced acute lung injury [50]. Plasma MIF increased significantly in pediatric patients undergoing surgery for congenital heart disease [51]. We found that additional mechanical stress further enhanced their release, both in the cold as well as the warm ischemia group. 

HMGB1 as well as MIF were released at earlier time points after liver explantation and graft ischemia compared to liver enzymes. During this interval, a statistically significant increase in AST and ALT was not observed.

## 5. Conclusion

AST and ALT as well as HMGB1 and MIF levels in the effluent can be used to assess the extent of damage from warm and cold ischemia in the liver graft. Notably, at early time points, additional mechanical stress led to a significantly higher release of HMGB1 and MIF but not of liver enzymes into the effluent than ischemia only. In this situation, a significantly increased nuclear cytoplasmic translocation of HMGB1 was visualized in hepatocytes by immunohistochemistry. Our results suggest that determination of HMGB1 and MIF reflects the extent of ischemic injury. In case of additional mechanical damage, as inflicted on the organ during surgical manipulation, additional determination of danger signals seemed to be superior to determination of liver enzymes only, as damage was indicated earlier.

## Figures and Tables

**Table 1:** Group distribution.

Damage type	Strain	Observation interval	Observation time	Effluent	Liver tissue
CI	Lewis (*n* = 6)	1 h	24 h	HMGB1; MIF	Histology
M+CI	Lewis (*n* = 6)	1 h	24 h	AST; ALT	HMGB1 IHC
WI	Lewis (*n* = 6)	0,5 h	6 h		
M+CI	Lewis (*n* = 6)	0,5 h	6 h		
CI	BN (*n* = 6)	1 h	24 h		

CI, cold ischemia; WI, warm ischemia; M, mechanical stress; HMGB1, high mobility group box-1; MIF, macrophage migration inhibitory factor; IHC, immunohistochemistry; AST, aspartate aminotransferase; ALT, alanine transaminase.
