# Corrigendum to “Release of Danger Signals during Ischemic Storage of the Liver: A Potential Marker of Organ Damage?”

**DOI:** 10.1155/2022/9870324

**Published:** 2022-05-09

**Authors:** Anding Liu, Hao Jin, Olaf Dirsch, Meihong Deng, Hai Huang, Martina Bröcker-Preuss, Uta Dahmen

**Affiliations:** ^1^Experimental Transplantation Surgery, Department of General, Visceral and Vascular Surgery, Friedrich Schiller University Jena, Drackendorfer Str.1, 07747 Jena, Germany; ^2^The Centre for Molecular Medicine, Shaoxing People's Hospital, 312000 Shaoxing, China; ^3^Department of General, Visceral and Transplantation Surgery, University Hospital Essen, University of Duisburg and Essen, 45122 Essen, Germany; ^4^Institute of Pathology, University Hospital Jena, 07747 Jena, Germany; ^5^Department of Clinical Chemistry, Clinic of Endocrinology, University Hospital Essen, University of Duisburg and Essen, 45122 Essen, Germany

In the article titled “Release of Danger Signals during Ischemic Storage of the Liver: A Potential Marker of Organ Damage?” [1], the authors identified an error in Figure 1 (a3 and a4). This error was introduced during the production of the publication , and the corrected figure is as follows:

## Figures and Tables

**Figure 1:**
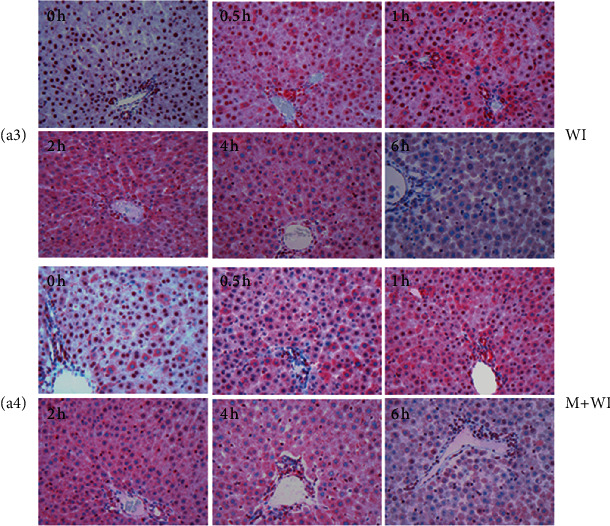
Immunohistochemical analysis of HMGB1 expression in rat livers following cold/warm ischemia. Staining pattern changed upon ischemia and mechanical stress (a). (a3) warm ischemia (WI), (a4) mechanical stress plus warm ischemia (M+WI).

## References

[1] Liu A., Jin H., Dirsch O. (2010). Release of danger signals during ischemic storage of the liver: a potential marker of organ damage?. *Mediators of Inflammation*.

